# Weighted Gene Co-Expression Network Analysis (WGCNA) Reveals the Functions of Syndecan-1 to Regulate Immune Infiltration by Influenced T Cells in Glioma

**DOI:** 10.3389/fgene.2022.792443

**Published:** 2022-05-20

**Authors:** Jiacheng Zhong, Shuang Shi, Wen Peng, Bei Liu, Biao Yang, Wenyong Niu, Biao Zhang, Chuan Qin, Dong Zhong, Hongjuan Cui, Zhengbao Zhang, Xiaochuan Sun

**Affiliations:** ^1^ Department of Neurosurgery, The First Affiliated Hospital of Chongqing Medical University, Chongqing, China; ^2^ Department of Neurosurgery, The People’s Hospital of Dazu, Chongqing, China; ^3^ State Key Laboratory of Silkworm Genome Biology, Southwest University, Chongqing, China; ^4^ Cancer Center, Medical Research Institute, Southwest University, Chongqing, China

**Keywords:** syndecan-1, glioma, immune infiltration, WGCNA, tumor microenvironment

## Abstract

Our previous studies shown that syndecan-1 (SDC1) may be a novel class of biomarkers for the diagnosis and treatment of glioma, but its specific roles and the in-depth molecular mechanism remain elusive. Here, we used Estimation of STromal and Immune cells in Malignant Tumor tissues using Expression data (ESTIMATE) algorithms and single-sample Gene Set Enrichment Analysis (ssGSEA) algorithms to evaluate the immune score of tumor samples and quantify the relative infiltration of immune cells in the tumor microenvironment (TME), respectively, in different data sets obtained from the Chinese Glioma Genome Atlas and The Cancer Gene Atlas. Next, we calculate the correlation of the immune score and immune cells with SDC1, respectively. To identify the specific process regulated by SDC1, the differentially expressed genes (DEGs) analysis between the high and low expression of SDC1 of glioma samples were used to discover the hub genes through Weighted Gene Coexpression Network Analysis (WGCNA). Gene Ontology (GO) and Kyoto Encyclopedia of Genes and Genomes (KEGG) analysis revealed cardinal biological processes and pathways involved in genes and tumor grade correlation and survival analysis verified its significance in glioma. The results show that SDC1 is associated with the immune infiltration of glioma in the TME, especially activated CD4+T cells and CD8+T cells. The three data sets filter 8,887 DEGs, the genes in the blue modules were selected as hub genes in WGCNA. GO and KEGG analysis found eight genes in the blue modules involved in antigen processing and presentation in T cells in glioma. Kaplan–Meier estimator and log-rank test statistic determined that the introduced genes are associated with poor prognosis in glioma. Protein–protein network interaction analysis showed that SDC1 may regulate antigen processing and presentation through CTSL or CD4 in glioma. Finally, this study provided insights and clues for the next research direction of SDC1 and identified the key pathways and genes that might participate in the immune escape of glioma. These results might provide a new insight on the study of immune infiltration of glioma in the future.

## Introduction

Glioblastoma multiforme (GBM) is one of the most common primary brain tumors in adults, and it has the characteristics of high invasion and poor prognosis (Tan et al., 2020). Despite extensive studies, the treatment of GBM has not improved much; the incidence rate associated with gradual decline in neurological function and quality of life may have devastating effects on patients, caregivers, and families (Taphoorn et al., 2010). The tumor microenvironment (TME) is becoming a pivotal regulator of cancer progression in primary and metastatic malignancies, especially in the immune context. For the brain, the number of these remarkable features, including the types of brain resident cells, blood–brain barrier, and immunosuppressive environment, constitute the unique characteristics of the organ (Quail and Joyce, 2017). Therefore, the additional investigations of immune infiltration in human glioma are urgently needed.

Syndecans is an evolutionarily conserved type I transmembrane protein family, lacking a common molecular structure (Leonova and Galzitskaya, 2013) and binding to various ligands and receptors, including transforming growth factor-*β*(TGF-*β*), platelet-derived growth factors (PDGFs), vascular endothelial growth factors (VEGFs), fibroblast growth factors (FGFs), integrin, and VEGFR (Rapraeger, 2000; Choi et al., 2011). They also regulate various signaling events both inside and outside cells. By binding to other molecules, syndecans play dual roles as both cell adhesion and docking receptors and participate in a variety of pathological processes, including cancer cell proliferation and invasion, angiogenesis, matrix remodeling, and host defense mechanisms (Kwon et al., 2012; Teng et al., 2012). Syndecan-1(SDC1) is the most widely studied member of the family. Studies show that the expression of SDC1 is different in different cancer types. The differential expression in stromal chamber and cancer cells is closely related to tumor invasiveness and clinical results (Akl et al., 2015; Gharbaran, 2015). Our previous research shows that the expression of SDC1 in human glioma is associated with advanced tumor progression and poor prognosis, and SDC1 knockdown inhibits glioma cell proliferation and invasion by deregulation of c-src/FAK related signal pathway (Shi et al., 2017).

Nowadays, with the research of TME, tumor immune infiltration is determined by the density, composition, functional state, and tissue of tumor leukocyte infiltration, which can produce information related to prognosis, prediction of treatment response, selection of treatment scheme, and various other pharmacodynamic parameters (Fridman et al., 2017). Therefore, the era of immunotherapy indicates the hope of developing more effective and better tolerated treatments to combat invasive diseases (Lim et al., 2018; Sampson et al., 2020), especially that reactivating the antitumor immune response can make the tumor subside through blockade of the checkpoint. However, this occurs only in a small number of patients with glioma. Studies of glioma reveal that glioma does not effectively respond to immunotherapy (Zappasodi et al., 2018). In the TME, the innate resistance prevents the initiation of immune response; adaptive immune resistance inactivates tumor-infiltrating immune cells, and acquired immune resistance protects tumors from being destroyed when attacked by the immune system (Jackson et al., 2019). Besides this, glioblastoma is considered to be an immune “desert” and a lack of infiltration by immune effector cells (e.g., CD8+T) (Domingues et al., 2016; Sokratous et al., 2017; Zhang et al., 2017). In addition, a limited number of T cells infiltrated the tumor, differing substantially from “self,” generally no response to checkpoint blockade (Zappasodi et al., 2018). Therefore, increasing tumor immunogenicity may be a key factor to improve immunotherapy.

Recent research shows that Syndecan-1 has roles variably in the regulation of TME (Handra-Luca, 2020), and the shed of SDC1 not only acts as an autocrine factor in the TME to stimulate tumor cell migration and angiogenesis, but also as a novel paracrine immunosuppressive mechanism to promote the extravasation and survival of malignant tumors (Jung et al., 2019). More and more studies show that SDC1 can help hosts escape immune surveillance. Oisun Jung et al. found that coupling of VEGFR2 to VLA-4 by shed Sdc1 inhibits LFA-1-mediated migration of T cells (Jung et al., 2019). Anil Kumar Jaiswal found that the combination of syndecan-1 and innate-like T cells may protect the hosts from autoimmune effects of interleukin-17 in autoimmune diseases (Jaiswal et al., 2018). Zhang et al. found that syndecan-1 negatively regulates initial recruitment of leukocyte into the brain across the choroid plexus in experimental autoimmune encephalomyelitis in mice (Zhang et al., 2013). Contrary to these phenomenon, Lotten Ragnarsson et al. found that multiple myeloma cells are killed by syndecan-1-directed superantigen-activated T cells (Ragnarsson et al., 2001). Generally speaking, Syndecan-1 might regulate the immune infiltration in the TME of glioma.

In this study, to investigate whether SDC1 affects the immune escape of glioma, we assess the correlation between SDC1 expression and immune scores in three data sets, and the correlation between SDC1 expression and specific immune cells was calculated. Second, we identified the differentially expressed genes (DEGs) between the high and low expression of SDC1, and find a hub module with a high correlation with SDC1 and immune scores with the WGCNA algorithm, and then we find the hub genes in the hub module. With Gene Ontology (GO) Enrichment Analysis and Kyoto Encyclopedia of Genes and Genomes (KEGG), we find the genes with high correlation with T cells and the signaling pathway. Protein–protein network interaction analysis (PPI) was used to analyze the interaction between SDC1 and hub genes. At last, we indent relevance through the survival analysis.

## Materials and Methods

### Study Design

**FIGURE 1:**
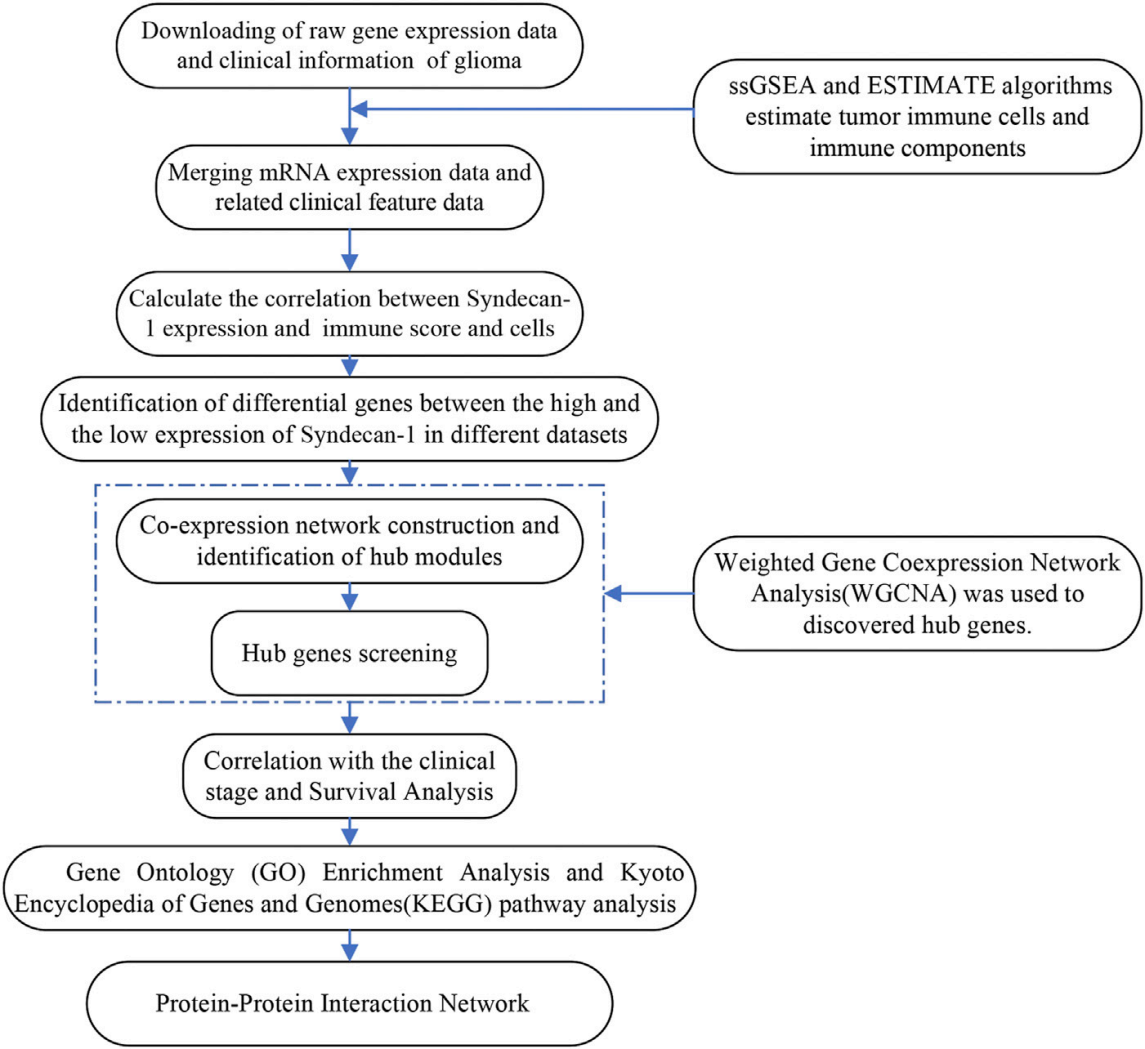
Flow chart describing the workflow from collection and preparation of the data to the data analyses.

This was an observational study using transcriptomic data of glioma in TCGA and CGGA. Based on our previous study of the function of SDC1 in glioma, our goal is to further explore the function of SDC1 in glioma. To study whether the expression of SDC1 in glioma is related to its immune infiltration, the samples in the data sets of glioma were evaluated through the comprehensive immune landscape of the TME through ESTIMATE algorithms. To ensure the reliability of the calculated immune landscape, the consistency of relevant clinical features with the immune landscape was evaluated. To find the specific immune cells that SDC1 may affect, the immune cell composition in the samples was also calculated by ssGSEA algorithms, the correlation of SDC1 between different immune cells was estimated. To find the genes that are not only related to SDC1, but also involved in glioma immune invasion, WGCNA was used to further data mining of differential genes of SDC1. After obtaining hub genes, GO and KEGG analysis was done to find the specific signal pathway. Finally, the reliability of the hub genes is evaluated by clinical prognosis analysis, and PPI predicts the possible mechanism of SDC1. Figure 1 illustrates the workflow chart of data preparation, processing, analysis, and validation.

### Data Acquisition and Processing

The raw gene expression levels and clinical information of glioma were downloaded from the Chinese Glioma Genome Atlas (CGGA) (http://www.cgga.org.cn, downloaded on 07/21/2021), which includes two data sets, mRNAseq_693 (*n* = 693) and mRNAseq_325 (*n* = 693). TCGA RNAseq and clinical information of glioma are downloaded from the TCGA database (https://portal.gdc.cancer.gov, downloaded on 07/21/2021, *n* = 702 after removing six duplicate samples). The leucocyte gene signature matrix refer to Charoentong et al., 2017, download from https://www.cell.com/cms/10.1016/j.celrep.2016.12.019/attachment/f353dac9-4bf5-4a52-bb9a-775e74d5e968/mmc3.xlsx).

### Identification of the Correlation Between SDC1 Expression and Immune Infiltration of Glioma

To visualize immune-related cell heterogeneity in the TME, two machine learning methods of gene expression profiles were used: the Estimation of STromal and Immune cells in Malignant Tumors using Expression data (ESTIMATE) (Yoshihara et al., 2013) and ssGSEA algorithms (Charoentong et al., 2017). First, the ESTIMATE algorithm was used to estimate the proportion of immune and stromal components in glioma samples. Spearman correlation analysis is used to assess the correlation between SDC1 expression and the degree of immune infiltration in glioma specimens. Second, to estimate the proportion of tumor-infiltrating immune cells (TICs), gene set variation analysis (GSVA) was used to estimate the variation of the immune cells through reference gene expression data set enrichment, that is, the leucocyte gene signature matrix (Charoentong et al., 2017); the proportions of 28 tumor-infiltrating lymphocytes were assessed in glioma tissues.

### Identification of DEGs Between the High and the Low Expression of SDC1

Based on the discovery that the expression of SDC1 increases with the increase of glioma grade, the tumor specimens with high and low expression of SDC1 were selected for differential analysis to study the possible function of SDC1 in glioma. After normalization of TCGA RNA-seq with the linear models for microarray data (LIMMA) package in the R language environment and log2 transformation, then the TCGA RNAseq, mRNAseq_693, and mRNAseq_325 were analyzed for the difference. The DEGs between the high and the low expression of SDC1 samples were identified (DEGs, *p* value < .05). The clustering heat map of differential genes is to assess the homogeneity of the samples. The Volcano maps are used to show genes that are significantly differentially expressed. Identification of unique genes within the DEG data sets in the three data sets, and the Venn diagram shows the overlapping results.

### Co-Expression Network Construction and Identification of Hub Modules

The WGCNA package is implemented in R (http://www.r-project.org/). WGCNA is an analysis method that analyzes gene expression patterns of multiple samples and is widely used in genome research. First, it is assumed that the gene network obeys a scale-free network, and the gene co-expression correlation matrix and the adjacency function formed by the gene network are defined, and then, the dissimilarity coefficients of different nodes are calculated, and a hierarchical clustering tree is constructed accordingly, which clusters genes with similar expression patterns. Different branches of the clustering tree represent different gene modules. The degree of gene co-expression in the module is high, and the degree of co-expression of genes belonging to different modules is low. Finally, the relationship between modules and specific traits or phenotypes was analyzed. Given the above algorithm, the expression of SDC1in glioma samples was selected as a clinical phenotype in our data mining. After removing outlier samples and those with incomplete clinical information, the adjacency matrix was constructed with 145 GBM samples. The soft thresholding power *β* was set to eight to ensure a correlation coefficient close to .9. A topological overlap matrix (TOM) plot was assessed as independent of each module. Module membership (MM) refers to the correlation coefficient between genes and module eigengenes and is used to describe the reliability of a gene belonging to a module. Finally, we calculated the correlation between the modules and the clinical phenotype, the modules that are not only related to SDC1, but also tumor immune infiltration, are selected to be the hub module.

### Hub Genes Screening

The intro module connectivity reflects the importance of nodes; it represents the sum of the degree of correlation between this gene and other genes in that module. For each expression profile, gene significance (GS) was calculated to assess the correlation between each gene and each trait; MM represents the correlation between a single gene and its module, indicating whether it is consistent with the trend of the module. The top 50% of genes with the highest intro module connectivity, GS, and MM were selected as hub genes in the hub modules.

### GO Enrichment Analysis and KEGG Pathway Analysis

To understand the biological meaning of the hub genes, the cluster profile (R package) was used for performing GO enrichment and KEGG pathway analyses (*p*-value <.05 was regarded as significant). The genes related to immune cells and immune signaling pathways are selected as target genes.

### Correlation With the Clinical Stage and Survival Analysis

To verify the importance of the expression of the key gene, the survival package was loaded in R and *survminer* was used for the survival analysis; 696 glioma tumor cases in TCGA had a detailed clinicopathological grading and survival time record, which were used for clinical correlation analysis. We evaluated the correlation between the target genes and clinical grade in the data set of glioma in the TCGA. The Kaplan–Meier method was used to plot the survival curve, and log-rank as the statistical significance test; *p* < .05 was considered significant.

### SDC1 and Hub Genes’ PPI Network

GeneMANIA (http://www.genemania.org) is a flexible, user-friendly web interface, which is used to generate assumptions about gene function, analyze gene lists, and determine gene priority for functional analysis (Szklarczyk et al., 2011). STRING is a database of known and predicted interactions, including direct (physical) and indirect (functional) associations from various sources, including high-throughput experiments and genomic contexts (Warde-Farley et al., 2010). SDC1 and Hub Genes were submitted to the above two websites to analyzes the PPI network.

## Results

### SDC1 is Associated With Immune Infiltration in Glioma

**FIGURE 2:**
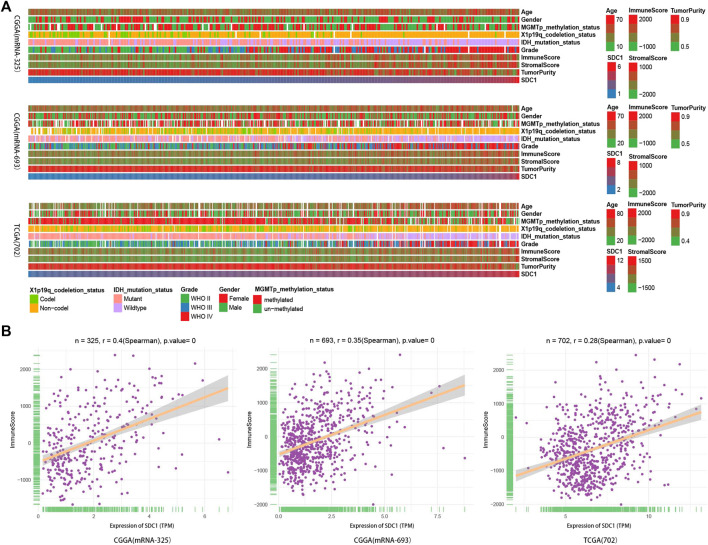
The landscape of glioma immune score, clinical and molecular characteristics in association with the expression of SDC1. **(A)** Transcriptome data set of glioma specimens in CGGA (mRNAseq_325 dataset and mRNAseq_693) and TCGA RNAseq were used to assess the immune score of glioma. **(B)** The relationship between the expression of SDC1 and glioma immune score was estimated. The patients’ characteristics of glioma further ensure the reliability of landscape of the expression of SDC1 and immune score. The association between the immune score in glioma samples and continuous variables was assessed using Spearman correlation tests.

To explore the correlation of SDC1 expression with immune infiltration, we first used the ESTIMATE algorithm to assessed glioma purity as well as stromal and immune scores. The landscape of clinical and molecular characteristics is described in three data sets, respectively (Figure 2A). As our previous reports show, the expression of SDC1 increased with the increase of tumor grade. Consistent with recent studies, Stromal and immune scores significantly increased along with malignancy progression, whereas glioma purity decreased in higher grades (Zhang et al., 2017). The IDH mutation and 1p/19q codeletion take place more in the low-grade gliomas, but the MGMT promoter mutation did not display this rule. Besides this, the incidence rate of men is higher than that of women. Most important of all, we found that SDC1 expression is a positive correlation with immune scores (Figure 2B). The correlation coefficient between SDC1 and immune scores is .4, .35, and .28 in the mRNAseq_325, mRNAseq_693, and TCGA RNAseq data sets, respectively.

### SDC1 is Associated With Activated CD4+T Cell and CD8+T Cell

**FIGURE 3:**
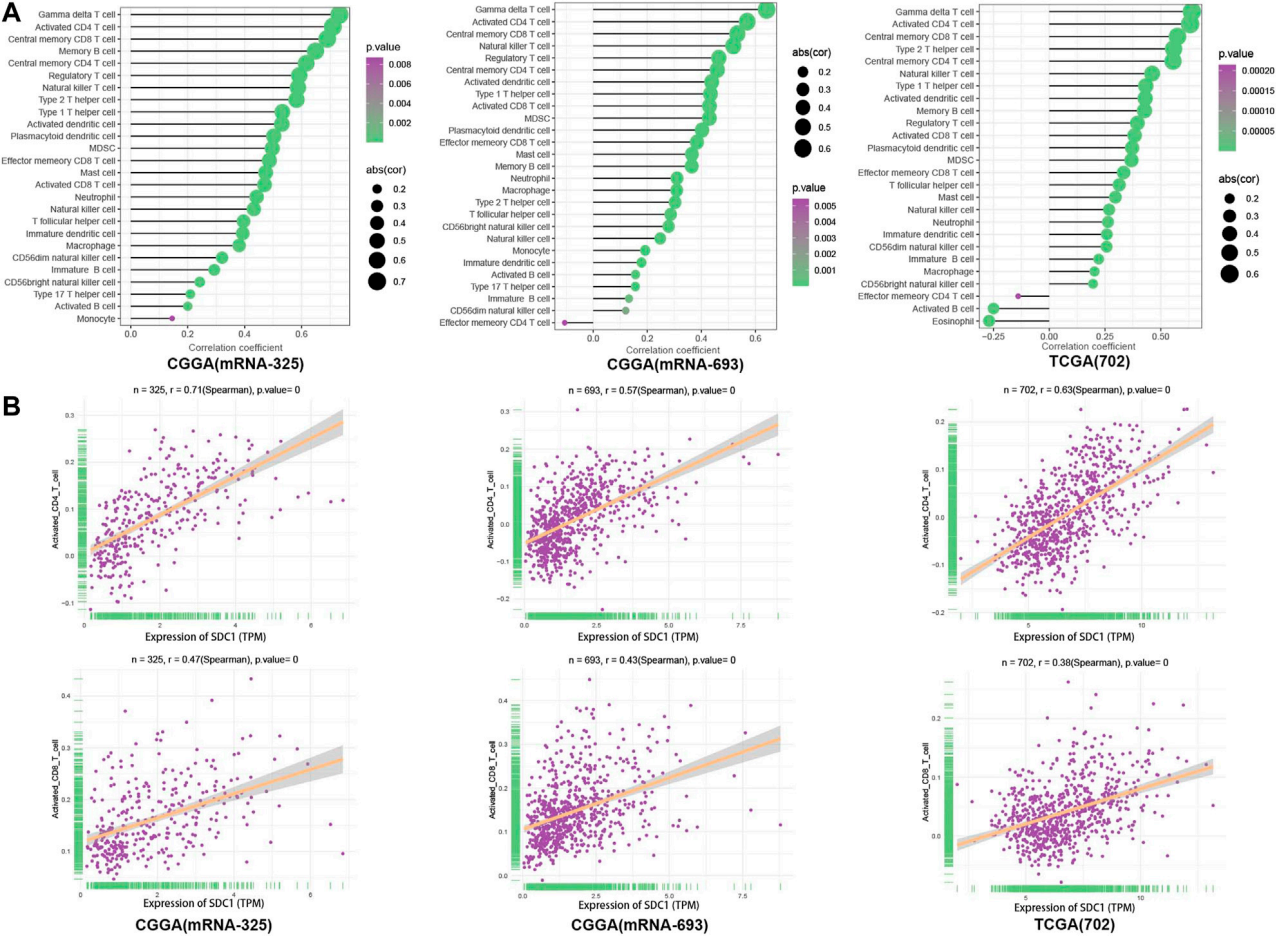
The TICs were evaluated through GSVA with ssGSEA algorithms; **(A)** the expression data set representing the leucocyte gene signature matrix was used as a reference; the proportions of 28 tumor-infiltrating lymphocytes were assessed in glioma tissues. **(B)** The relationship between the immune cells and the expression of SDC1 in glioma samples was assessed using Spearman correlation tests.

The ssGSEA was introduced to quantify the relative infiltration of 28 immune cell types in the TME in the three data sets. We calculate the correlation of SDC1 with each immune cell (Figure 3A); the correlation coefficient between SDC1 and activated CD4+T cells is .71, .57, and .63 in mRNAseq_325, mRNAseq_693, and TCGA RNAseq data sets, respectively. The correlation coefficient between SDC1 and activated CD8+T cell is .47, .43, and .38 in mRNAseq_325, mRNAseq_693, and TCGA RNAseq data sets, respectively (Figure 3B).

The DEG analysis between the high and low expression of SDC1 of glioma samples.

**FIGURE 4:**
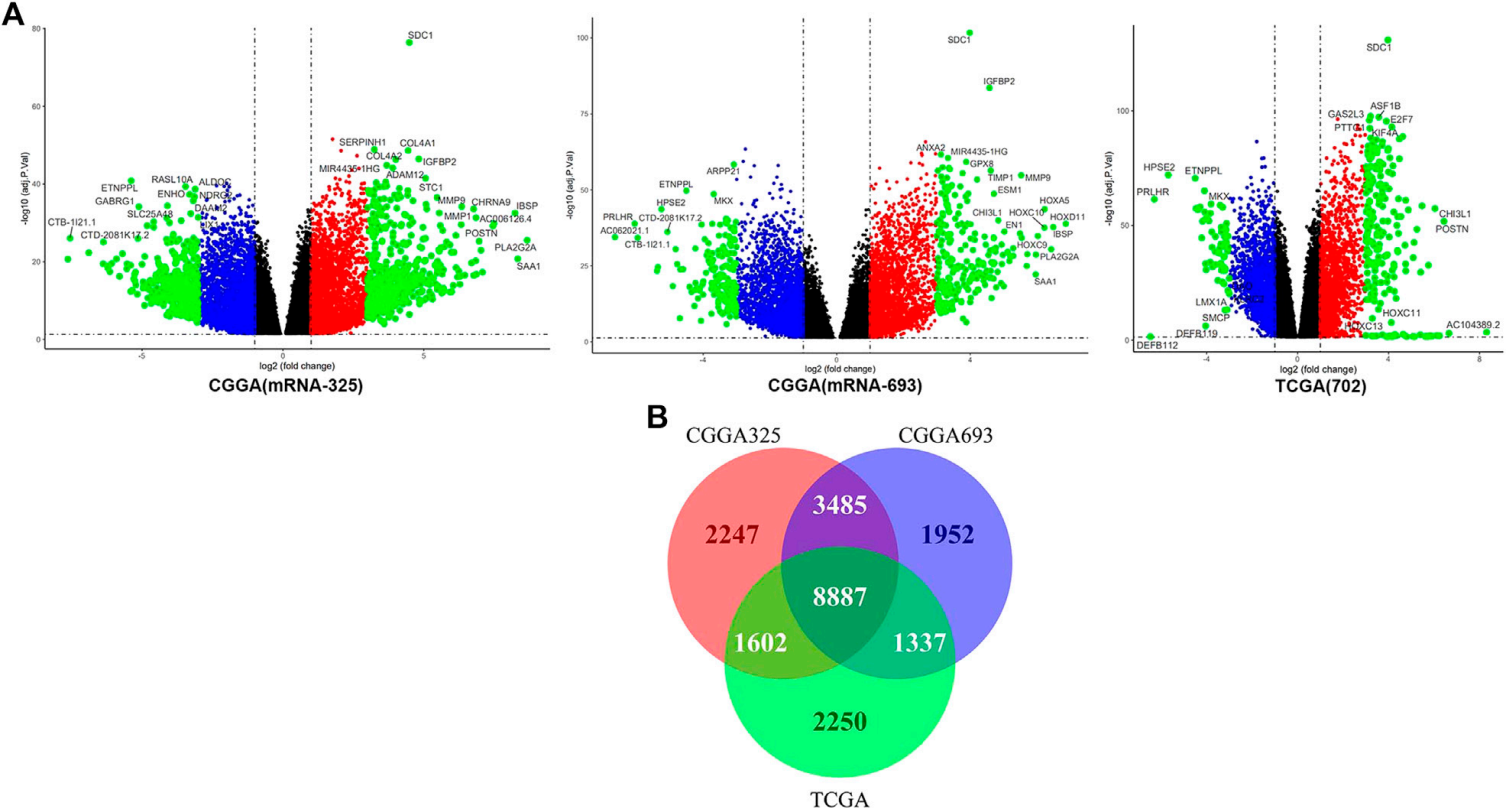
**(A)** The volcano maps display the differential genes. **(B)** The Venn diagrams reveal the coexisting genes in the three different gene sets; there are 8,887 genes in the coexist sets.

The DEGs in the three data sets were calculated though the LIMMA package. The best homogeneity between tumor samples in the high and low expression of SDC1 of glioma samples is shown in the TCGA data sets. The volcano maps show the multiple changes of different genes more clearly (Figure 4A). The black points represent the genes that abs (logFc) < 1, p-value < .05; the red and blue points represent the genes which 1 < abs (logFc) < 3, p-value < .05; and the green points represent the genes which 3 < abs (logFc), p-value < .05. To get more reliable differential genes within the DEG data sets, the unique genes sets were determined using Draw Venn Diagram (Figure 4B). The number of 8,887 genes was selected to WGCNA in TCGA-seq data sets in the next data analysis.

### Identification Hub Gene Modules by WGCNA

**FIGURE 5:**
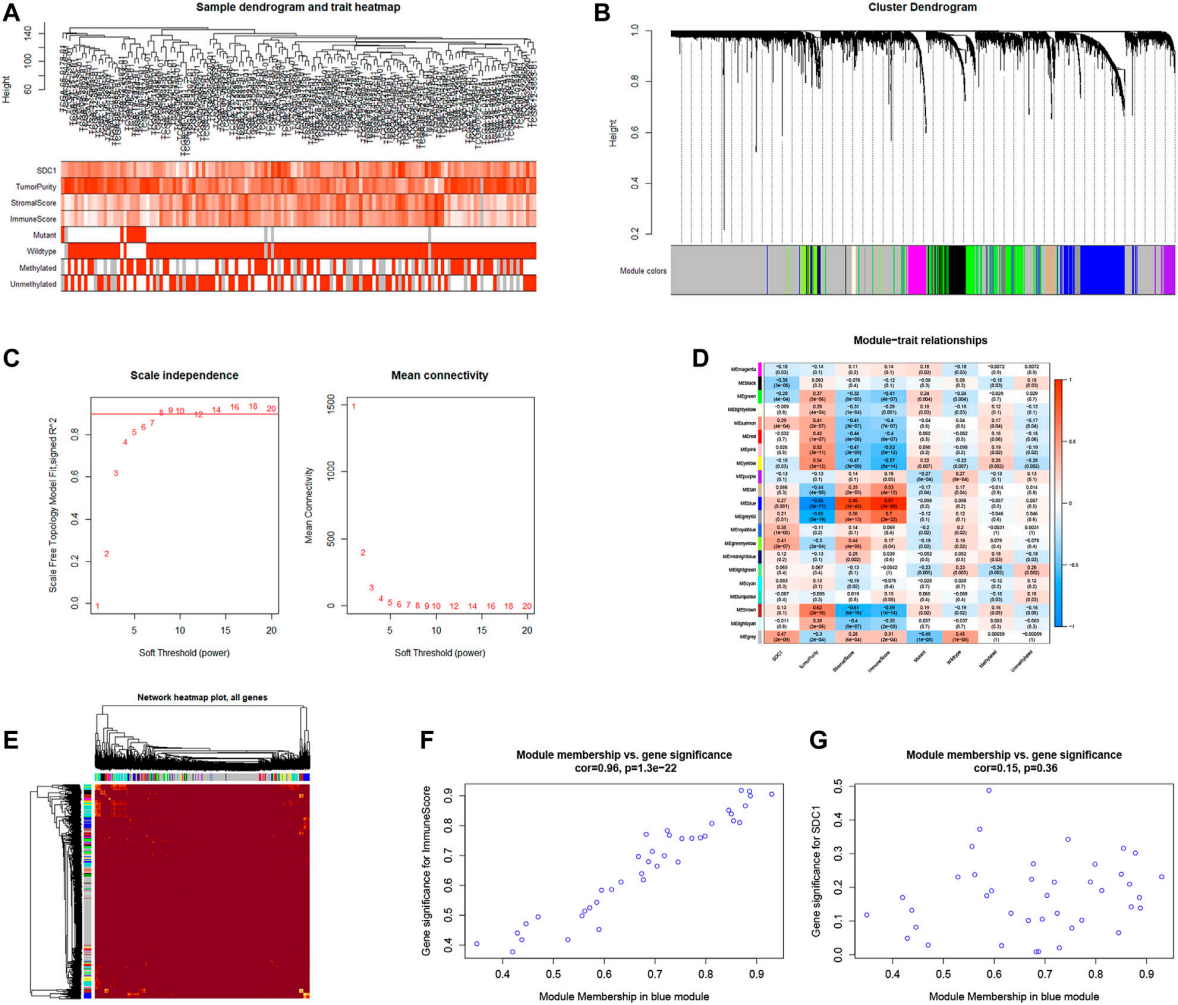
The WGCNA method was used to find the genes that are not only related to SDC1 but also involved in glioma immune infiltration. **(A)** Mapping of clinical trait variables and aggregation trees show the landscape of the expression of SDC1, immune score, and clinical phenotype. **(B)** The dynamic tree display different color-coded co-expression modules were constructed. **(C)** The soft thresholding and correlation coefficient in the scale-free topology fitting graph. **(D)** Correlation between module eigengenes and clinical traits. The clinical traits include the expression of SDC1 and immune score. The corresponding correlations and *p*-values are presented. **(E)** A TOM plot showed that each module in the network was independent of each others, further indicating that gene expression in each module was also relatively independent. **(E,F)** The blue module was identified to have the highest positive correlation with the expression of SDC1 and immune score.

The GBM samples with clinic characteristics were selected to find the gene sets not only related to SDC1 but also tumor immune infiltration. After removing outlier samples, 145 samples were used to construct an adjacency matrix (Figure 5A). Mapping of clinical trait variables and aggregation trees display that WGCNA analysis can be performed. The dynamic tree display different modules were constructed (Figure 5B). The soft thresholding power *β* was set to eight to ensure a correlation coefficient close to .9 (Figure 5C). A total of 22 different color-coded co-expression modules were identified (Figure 5D). A TOM plot showed that each module in the network was independent of each other, further indicating that gene expression in each module was also relatively independent (Figure 5E). After combining the module conservatism test, the Z-scores >10 of the module were selected. No modules are excluded. Subsequently, we analyzed the module related to both SDC1 and immune invasion in glioma. The blue module (contained 685 genes) was significantly correlated with immune scores and correlates with SDC1; therefore, this module was selected as a significant module for further analyses. Besides this, we painted scatterplots of MM versus gene significance (GS) of SDC1 and immune scores, and a correlation was obtained between the blue module and SDC1 (Figures 5F,G). The genes of the hub modules with the top 50% intro module connectivity, MM, and GS were selected as hub genes.

### GO and KEGG Analysis for the Module Gene

**FIGURE 6:**
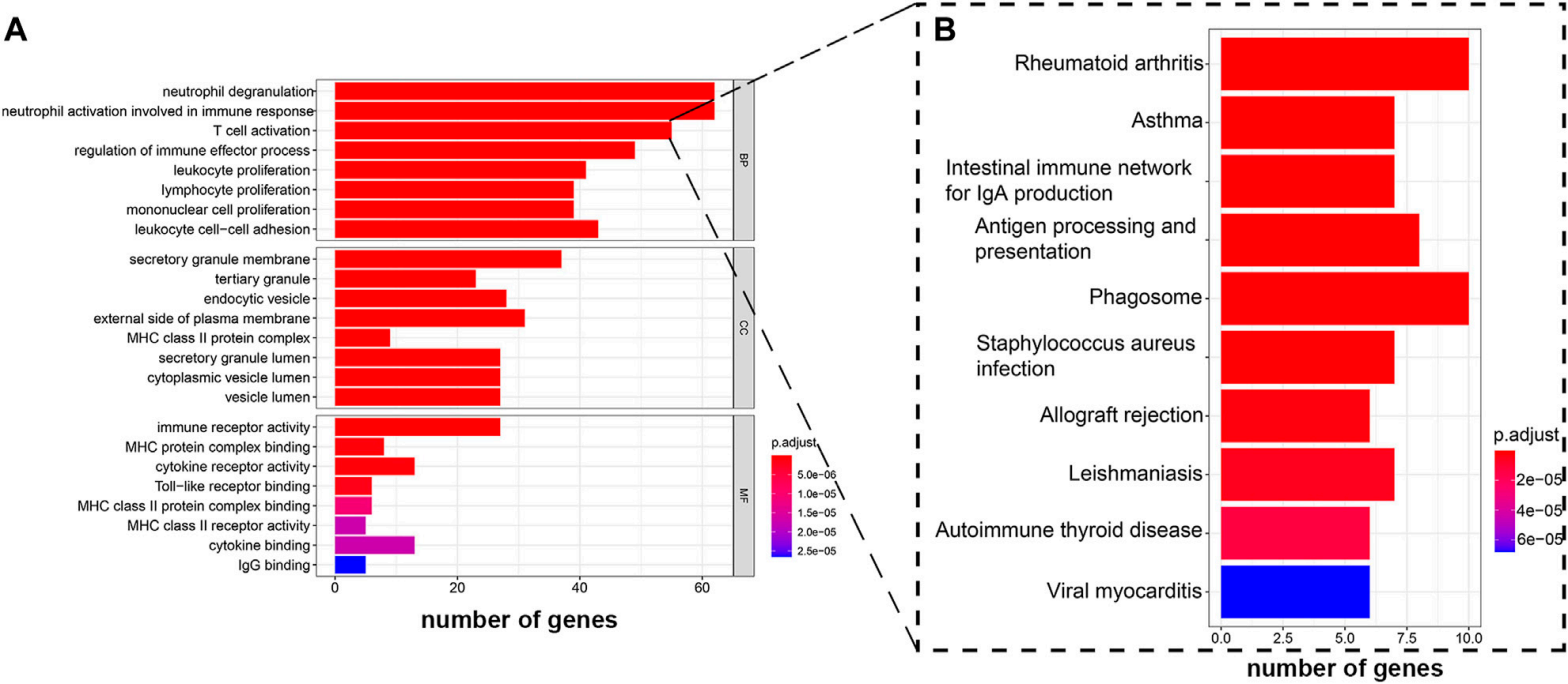
GO enrichment analysis and KEGG pathway analysis were performed on hub genes. **(A)** GO enrichment analysis shows the hub genes participate in T cell activation. **(B)** KEGG pathway analysis displays the genes that activated T cells participated in antigen processing and presentation.

The hub genes in the blue module were selected for GO analysis, the BP group genes were mainly associated with activation of lymphocytes, and MF group genes were mainly enriched in the binding of antigen and antibody. We selected the group genes that correlated with T cells as target genes, which include 55 genes (Figure 6A). To explore potential signaling pathways relating SDC1 to immune scores, KEGG pathways were employed to identify the function of the signaling pathway. We found eight genes correlated with antigen processing and presentation (Figure 6B). The eight genes are CTSL, HLA-DPA1, HLA-DPB1, HLA-DRB1, CD74, HLA-DOA, HLA-DMB, and CD4.

### The Target Genes Were Significantly Correlated With Clinical Grade and Prognosis of Glioma

**FIGURE 7:**
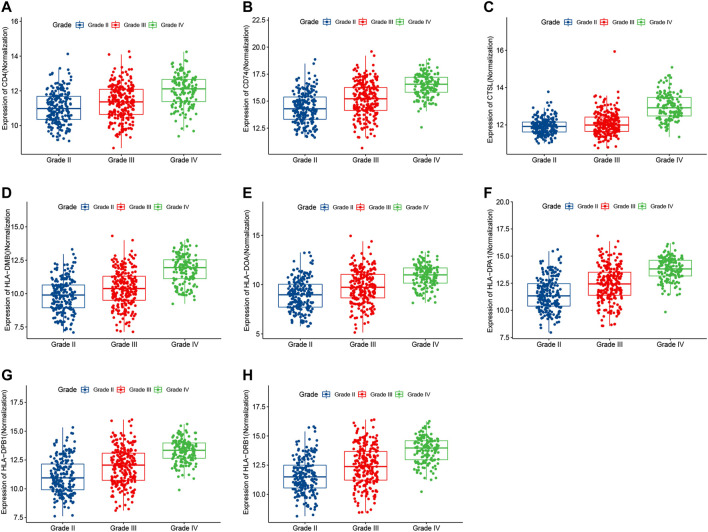
The expression levels of target genes were correlated with more advanced tumor grades in TCGA (Kruskal–Wallis nonparametric test, *p* < .0001).

**FIGURE 8:**
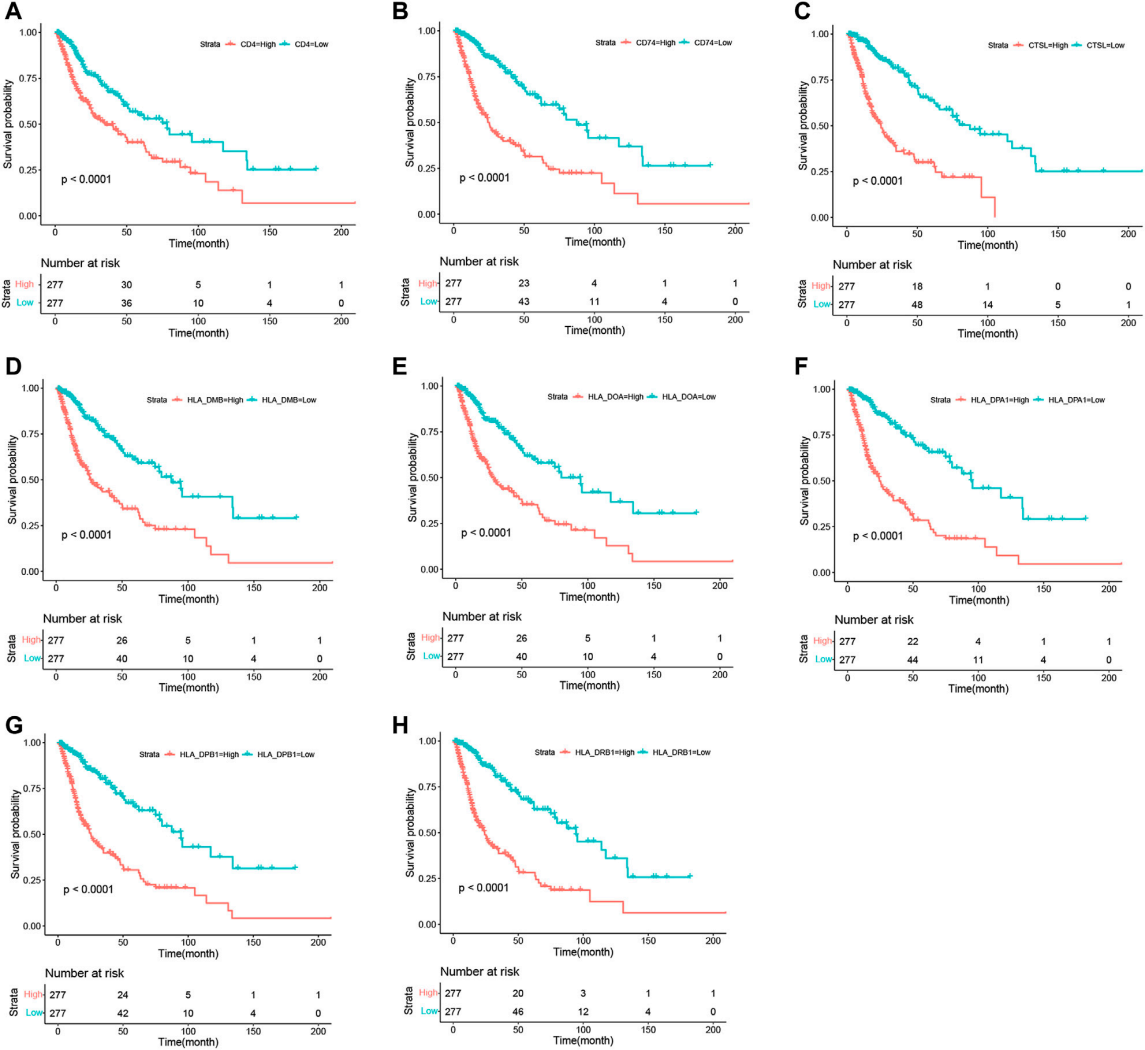
Kaplan–Meier survival curves in glioma patients in TCGA show that the expression levels of target genes predicted prognosis. High expression: SDC1 levels in the upper median; Low expression: SDC1 levels in the bottom median.

To determine the associations between target genes in human glioma and increases in tumor progression and poor prognosis, the LGG and GBM data from TCGA data sets were analyzed. The target gene expression levels (Figures 7A–H) were significantly correlated with more advanced tumor grades (Kruskal–Wallis nonparametric test, *p* < .0001 for both). Moreover, analysis of TCGA patient survival revealed that elevated target gene expression predicted prognosis (Figures 8A–H). Glioma patients with a high level of target genes above the median had dramatically decreased survival compared with those with target gene levels below the median (Kaplan–Meier survival analysis, *p* < .0001 in log-rank). These data confirm that target genes play a crucial role in the tumorigenesis of glioma.

### SDC1 and Hub Genes’ PPI Network

**FIGURE 9:**
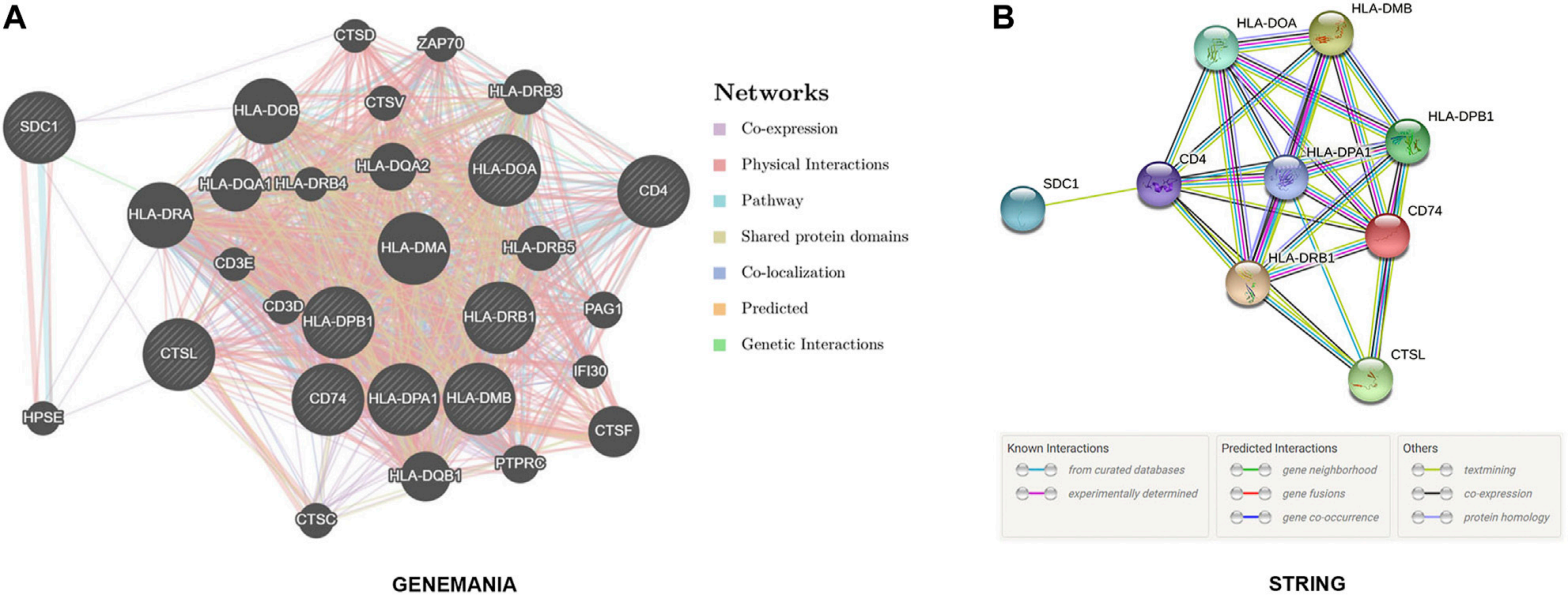
GeneMANIA (https://genemania.org) and String’s online database (https://string-db.org) reveal that SDC1 may interact with CD4, CTSL, and HPSE directly or indirectly involved in the regulation of T cell activation.

To explore the protein interaction between SDC1 and the target genes as well as the interaction with other genes, the GeneMANIA (https://genemania.org) and String online database (https://string-db.org) was used to perform PPI analysis, and the results showed that SDC1 may interact with CD4, CTSL, and HPSE directly or be indirectly involved in the regulation of T cell activation (Figure 9).

## Discussion

Glioma tissues consist of not only glioma cells but also glioma-associated nontumor cells, such as stromal and immune cells, which are important components of the TME. Recent research shows that TME plays a crucial role in the initiation and progression of glioma. An important component of the progression of glioma is communication with and manipulation of other cells in the brain environs (Broekman et al., 2018). GBM cells not only suppress resident immune cells and change their phenotype to support tumor growth, but also inhibit recruited immune cells. Consequently, immune cells play an important role in the occurrence and development mechanism of glioma. Therefore, increasing understanding of how immune cells access the brain and how the tumor thwarts the immune response offers new strategies for mobilizing an antitumor response.

Our previous research shows that SDC1 expression in human glioma correlates with advanced tumor progression and poor prognosis, and SDC1 knockdown inhibits glioma cell proliferation and invasion by deregulating a c-src/FAK-associated signaling pathway (Shi et al., 2017). SDC1 might regulate tumor cell immune invasion in the TME and even help tumor cells escape immune surveillance (Jung et al., 2019). To further study the function of SDC1 in the glioma microenvironment, we analyzed the possibility of SDC1 related to immunity at the transcriptome level in different databases and searched for the possible molecular and signaling pathway.

Nowadays, immunoscore provides a broad overview of the main immune parameters positively shaping cancer development and correlated with their prognostic and predictive value (Bruni et al., 2020). ESTIMATE provides researchers a tool for predicting tumor purity and the presence of infiltrating stromal/immune cells in tumor tissues using gene expression data. The ESTIMATE algorithm is based on single sample GSEA and generates three scores: 1) stromal score (which captures the presence of stroma in tumor tissue), 2) immune score (which represents the infiltration of immune cells in tumor tissue), and 3) estimate score (which infers tumor purity) (Yoshihara et al., 2013). ssGSEA, an extension of GSEA, calculates separate enrichment scores for each pairing of a sample and gene set (Subramanian et al., 2005). Combined with immune genes (Charoentong et al., 2017; Finotello and Trajanoski, 2018), the ssGSEA enrichment analysis was used to quantify the degree of immune infiltration within a sample in the different data sets. After evaluation by ESTIMATE and ssGSEA algorithms, the landscape of clinical and molecular characteristics is described in three data sets, respectively (Zhang et al., 2017) (Figures 2, 3). We found that SDC1 expression is a positive correlation with immune scores (Figure 2B). The correlation coefficient between SDC1 and immune scores suggests that SDC1 may play a role in regulating immune infiltration in the tumor microenvironment of glioma. To find out which immune cells SDC1 may regulate, we used the ssGSEA algorithm in three data sets to quantify the relative invasion of 28 immune cells in the TME. We calculate the correlation of SDC1 with each immune cell (Figure 3); the results show that SDC1 is associated with activated CD4+T and CD8+T cells. It suggests that SDC1 may have a positive correlation with activated CD4+T and CD8+T cells in the TME of glioma.

SDC1 is an evolutionarily conserved family of type I transmembrane proteins that lack a common molecular structure; it functions through binding various ligands and receptors. How is it involved in activating T cells? First, we identified the DEGs between the high and low expression of SDC1 samples in the mRNAseq_325, mRNAseq_693, and TCGA RNA-seq data sets. The Venn diagrams were generated to dig more reliable differential genes within the DEG data sets (Figure 4B). The number of 8,887 different genes was obtained by drawing the Venn diagram. To find the downstream genes related to both SDC1 and immune infiltration in glioma, WGCNA was used to analyze the overlap differential genes in the TCGA-seq data set. WGCNA is a new systems biological method (Gu et al., 2019) based on microarray or RNAseq data that is increasingly used to discover the relationship between networks, genes, and phenotypes (Wei et al., 2020). WGCNA transformed gene expression data into co-expression modules, providing insights into signaling networks that might be responsible for phenotypic traits of interest (Wan et al., 2018) and provides a systems-level insight, high sensitivity to low abundance, or small fold change genes without any information loss (Pei et al., 2017). Recently, studies have shown that WGCNA oﬀered important modules and pathways of many diseases (Guo et al., 2019; Wang et al., 2020; Wei et al., 2020).

Subsequently, we analyzed the module related to both SDC1 and immune infiltration in glioma (Figure 5D). The blue module, which contained 685 genes, was selected as the hub module. The top 50% genes of the hub modules with the highest intro module connectivity, MM, and GS were selected as hub genes. Fifty-five genes in the blue module were found involved in the activation of T cells through the GO enrichment analysis. To find out how these genes affect the activation of T cells, KEGG analysis was used to analyze the 55 genes; we found eight genes involved in antigen processing and presentation, including CTSL, HLA-DPA1, HLA-DPB1, HLA-DRB1, CD74, HLA-DOA, HLA-DMB, and CD4. Maybe they would be involved in the immune escape of glioma. Further, we evaluated the clinical significance of these eight genes to confirm their importance. It reveals that our target genes were significantly correlated with clinical grade and prognosis of glioma. Our study suggests that SDC1 may regulate the activation of T cells by affecting the antigen presentation of glioma cells. Further experiments are needed to elucidate the molecular mechanisms of antigen processing and presentation affected by SDC1 in the carcinogenesis of glioma, which may lead to a novel prognostic indicator and more effective treatment strategies.

The major limitation of this study is that the specific mechanisms affected by SDC1 have not been explored by the experiment. In future studies, we will experiment with cells and animals to verify and further explore upstream and downstream interactions of SDC1 in glioma.

## Data Availability

Publicly available data sets were analyzed in this study. This data can be found here: http://www.cgga.org.cn(CGGA); https: //portal.gdc.cancer.gov(TCGA).
